# Editorial: Long term disability in neurological disease: A rehabilitation perspective

**DOI:** 10.3389/fneur.2022.964664

**Published:** 2022-08-17

**Authors:** Alessio Baricich, Grazia F. Spitoni, Giovanni Morone

**Affiliations:** ^1^Physical and Rehabilitative Medicine, Department of Health Sciences, Scuola di Medicina, Università degli Studi del Piemonte Orientale, Novara, Italy; ^2^Azienda Ospedaliero Universitaria Maggiore della Carità, Novara, Italy; ^3^Department of Dynamic, Clinical Psychology and Health, Faculty of Medicine and Psychology, Sapienza University of Rome, Rome, Italy; ^4^Santa Lucia Foundation (IRCCS), Rome, Italy; ^5^Department of Life, Health and Environmental Sciences, University of L'Aquila, L'Aquila, Italy

**Keywords:** neurological disease, rehabilitation, chronic disease, long term rehabilitation, management - healthcare

Neurological diseases are often associated with a significant burden of disability, which can severely affect different aspects of patients' autonomy, notably motor and cognitive impairments. These impairments can arise in a progressive and long-term manner, as expected in neurodegenerative diseases and after acute conditions such as strokes, traumatic brain injuries, or spinal cord injuries. The clinical and social impact of these conditions is critical.

As outlined in the recent guidelines, stroke represents the second cause of mortality worldwide, drawing attention to improving the acute care of disease successfully, leading to a significant reduction in mortality (1).

However, due to this central focus, the long-term effects have been under-explored, leaving strokes a significant cause of disability. Even if strokes are generally considered and managed as a transient condition, most stroke survivors suffer from persistent critical limitations in the activities of daily living. 50% of stroke survivors report unmet needs such as incontinence, emotional problems, mobility, pain, and speaking problems. However, most of them do not receive a rehabilitative follow-up or other therapeutic approaches (2).

It is known that recovery is a complex process, which probably implies a combination of spontaneous and learning-dependent processes and adaptive behavior. Current evidence suggests that several mechanisms are involved, including restoring the functionality of damaged neural tissue (e.g., restitution), reorganization of spared neural pathways (e.g., substitution), improvement of impaired skills in the activities of daily living (e.g., compensation) (3) and last but not least, the recovery of cognitive skills.

Considering these aspects, there is cumulative evidence that interdisciplinary rehabilitation treatment improves the outcomes of stroke survivors when applied in acute and subacute phases after the event (4, 5). Indeed, the “formal” post-stroke motor rehabilitation usually ends 3–4 months after the event, based on the fact that motor and functional recovery reaches a debated plateau 3–6 months after stroke (6). However, current evidence supports the hypothesis that cognitive (Wang et al.; Rohrbach et al.) and motor skills may improve at any time after stroke, as well as in other pathologies such as other conditions that might critically affect the central nervous system (Cammisuli et al.; Elena et al.; Calafiore et al.) or muscular inherited muscular diseases (Alvarez et al.).

Brain plasticity phenomena are also widely involved in the chronic phase, albeit to a lesser extent than in the subacute phase. They lead to a modification of the cortical network, which can, in some cases, lead to clinically significant functional improvements. We know that rehabilitation may promote favorable neural plasticity (7, 8); notably, these processes may be reinforced by the use of innovative techniques and devices (Bressi et al.; Li et al.; Caimmi et al.; Peng et al.). In addition, the use of innovative orthoses and prostheses can reduce the impact that loss of function or organ damage has on the patient's abilities, improving their emotional state and consequently increasing social engagement (Pundik et al.).

However, future studies should focus on the development of a theoretical model to better understand the neurophysiological aspects of CNS recovery, as suggested by an interesting study protocol proposed by Simis et al.

In chronic stroke, modifications and possible modulations are linked not only to the brain and brain plasticity but also to the peripheral skeletal muscle in an interdependent way. Azzollini et al. discuss this topic in their review.

In addition, long-term unmet needs are observed in many domains, including social reintegration, health-related quality of life, maintenance of activity, and self-efficacy. From this point of view, stroke should be considered a chronic disease, and rehabilitation processes should be designed considering also these aspects. In this regard, rehabilitation services must have proper patient management in the form of a dedicated clinical pathway considering each individual's many different factors, including clinical, social, and economic aspects. In this line, identifying the target patient subgroup is the new challenge of translational medicine and, in particular, the rehabilitation that has high costs and is resource consuming. Studies that aim to identify prognostic factors, not only for conventional therapy but even for technologically assisted training, are essential to plan future effective rehabilitation plans (Wu et al.; Lee and Shin) or to identify subjects unable to return to work after a CNS lesion (Iosa et al.).

Additionally, some recent technology innovations may help patients' follow-up adherence. These aspects should be considered where the patient is unable to reach rehabilitation facilities or in low-income countries where outcomes are less favorable, as suggested by Contrada et al..

Technology is not the only answer to meeting patients' needs in a long-term perspective.

Current literature suggests the positive impact of peer support programs (9), and Baumgartner-Dupuits et al. proposed a study protocol to clarify these aspects.

In another intriguing study, Grimm et al. explored the potential impact of biographical music and biographical language on physiological responses and the endocrine system of people with disorders of consciousness.

From what has been briefly set out, a picture emerges in which an initial acute phase must necessarily be followed by a phase involving long-term interventions. In this phase, patient care must include an intervention in which the various professional figures together with territorial medical services must tune in and integrate to allow the patient the best possible quality of life.

## Author contributions

AB: conception and design of the paper and first draft of the manuscript. GM and GS: conception and design of the paper and manuscript and revision. All authors contributed to the article and approved the submitted version.

## Conflict of interest

The authors declare that the research was conducted in the absence of any commercial or financial relationships that could be construed as a potential conflict of interest.

## Publisher's note

All claims expressed in this article are solely those of the authors and do not necessarily represent those of their affiliated organizations, or those of the publisher, the editors and the reviewers. Any product that may be evaluated in this article, or claim that may be made by its manufacturer, is not guaranteed or endorsed by the publisher.
